# Factors associated with the survival of prostate cancer patients with rectal involvement

**DOI:** 10.1186/1746-1596-9-35

**Published:** 2014-02-20

**Authors:** HaiTao Wang, YanHong Yao, BaoGuo Li

**Affiliations:** 1 Department of Interventional Oncology, Tianjin Medical University Cancer Institute and Hospital, National Clinical Research Center for Cancer, Tianjin, China; 2 Research Group of Evidence-based Clinical Oncology, Tianjin, China; 3 Tianjin Key Laboratory of Cancer Prevention and Therapy, Tianjin, China

**Keywords:** Prostate cancer, Rectal involvement, Total pelvic extenteration, Prognostic factors

## Abstract

**Background:**

Prostate cancer patients with rectal involvement are rare, and the factors associated with the survival of these patients are yet to be elucidated.

**Patients and methods:**

We collected data on patients who were admitted to our hospital for prostate cancer in the last thirteen years and of those in studies in the literature. The associations of clinical characteristics with survival were evaluated using Cox regression models.

**Results:**

This study included 94 patients (5 admitted to our hospital and 89 from studies in the literature) of prostate cancer with rectal involvement. 11 patients in the group of synchronous rectal involvement at first cancer diagnosis (n = 58) and 23 patients in the group of metachronous diagnosis of rectal involvement (n = 29) died at the latest follow up. The estimated overall survival rate (% ± SE) at 1, 3, and 5 years were 68.3 ± 5.3%, 54.4 ± 7.2%, and 38.1 ± 11.1%, respectively. In the Cox univariate analysis, Asian prostate cancer (p = 0.001) was associated with better survival, while rectal bleeding (p = 0.043), metachronous presentation of development of rectal involvement (p = 0.000), prior hormonal therapy (p = 0.000) and extrarectal metastases (p = 0.054) were associated with poor survival. In multivariate analysis, prior hormone therapy (HR = 14.540, p = 0.000) and rectal bleeding (HR = 2.195, p = 0.041) retained independent poor prognostic values. There were 13 patients survived for more than 3 years, the longest survival time was 96 months. Total pelvic extenteration (TPE) combined with hormonal therapy in 12 hormone-untreated prostate cancer give us six of thirteen long-term survivors for more than 3 years in this series.

**Conclusions:**

Our findings suggest that rectal involvement does not necessarily predict a worse outcome when presenting as a previously hormone-untreated disease and that the prognosis was worse when presenting as a hormone relapsed disease. Prior hormone therapy and rectal bleeding were associated independently with a significantly poor overall survival in prostate cancer patients with rectal involvement. TPE combined with hormonal therapy appears to confer better overall survival in hormonally untreated patients.

**Virtual slides:**

The virtual slide(s) for this article can be found here: http://www.diagnosticpathology.diagnomx.eu/vs/1604504118106105.

## Introduction

Prostate cancer (PCa) is the most common malignancy in Western countries and the second leading cause of cancer-related deaths in males [1-3]. In China, the incidence of prostate cancer has increased dramatically over the past two decades, most likely due to economic development and lifestyle changes [4]. According to the recent statistics in China, PCa is the 7th most common cancer in males, and its incidence rose to 11 per 10^5^ in the year 2008 [5]. Despite improvements in diagnosis, surgical techniques, and chemotherapies, most deaths of PCa patients occur due to disease progression and metastasis.

Metastatic disease to the bones and lymph nodes has long been recognized as the most typical pattern of extraprostatic tumor spread [6]. Prostate cancer with rectal involvement has only rarely been reported and remains poorly understood [7-10].

In this study, we collected data on all patients who were admitted to our institution because of this rare tumor over the past 13 years, and thoroughly reviewed the literature for individually documented cases of prostate cancer with rectal involvement to comprehensively analyze patient and tumor characteristics, survival and prognostic factors in these rare tumors.

## Patients and methods

### Patient population

The patients examined in this study were enrolled in the following manner. First, we studied the data on all patients who were diagnosed with rectal involvement of prostate adenocarcinoma at the Tianjin Medical University Cancer Center and Institute between January 1990 and April 2013. These cases were retrospectively reviewed on the basis of histological examination of a surgical specimen or tissue biopsy. Rectal cancer patients with metastasis to the prostate or with synchronous and metachronous adenocarcinoma of the prostate and rectum were excluded. In all cases in our cohort, the diagnosis of prostate cancer with rectal involvement was determined by rectal biopsy and/or prostate biopsy or postoperative histology. Most of them were confirmed with immunohistochemical staining for prostate specific antigen (PSA) or prostatic acid phosphatase (PAP). This study was carried out in accordance with the principles of the Helsinki Declaration and approved by the Institutional Review Board of Tianjin Medical University Cancer Institute and Hospital. Written informed consent was obtained from all of the study participants.

Second, a systematic search of the literature was performed to identify all relevant articles dealing with prostate cancer with rectal involvement. First, a computerized search was made of the electronic databases PubMed (1990 to 2013) and google scholar (1990 to 2013) with the following Mesh headings and key words: “prostate/prostate neoplasms/prostate cancer” in combination with “rectum or rectal involvement or invading”. The search was exploded using the “related articles” term in PubMed. The abstracts of all identified articles were studied and selection was based on their relevance for the subject. The reference lists of selected articles were then screened systematically for additional studies of interest. No restrictions for language were applied. Articles containing duplications of case descriptions were excluded. In addition, patients with prostate special pathological type, including sarcoma, squamous cell carcinoma,small cell carcinoma and so on were excluded because of the different tumor biology in comparison with prostate adenocarcinoma. We reviewed the titles to include case reports or review articles on or case series of prostate cancer invading rectum. Altogether, 35 reference articles were suitable for further review. All 35 articles were reviewed to identify patients with prostate cancer invading rectum.

### Clinical data

Clinical data including patient demographics, clinical characteristics, extent of disease (extrarectal Pca metastases), type of rectal involvement, treatment modalities, and follow-up on these patients were extracted by 2 authors, then crosschecked to ensure accuracy, and finally entered into a database. Some factors had higher missing values because secondary data were collected in the literature review. Clinical data at our institute were determined by reviewing their medical records. Type of rectal involvement was defined according to the criteria described in the literature [11,12]. Overall survival was defined as the time from the date of diagnosis of prostate cancer with rectal involvement to the date of death. In the absence of confirmation of death, survival time was censored at the last date the patient was known to be alive.

### Statistical analyses

Data on patient demographics, tumor characteristics, extent of disease, treatment modalities were summarized using descriptive statistics. The associations between these factors and survival were evaluated using the univariate Cox regression models. The hazard ratio of death and its 95% confidence interval were calculated for each factor. Multivariate Cox proportional hazards regression was conducted to investigate independent predictors of survival. Variables were entered in the multivariable model when the P value was less than 0.05 in univariate Cox analysis and have complete follow up information. The median survival time was calculated using the Kaplan–Meier method. In order to present survival distribution by significant variables, survival curves were generated using the Kaplan–Meier method. Generally, p < 0.05 (two-sided) was considered statistically significant. Statistical analyses were performed with SPSS version 16.0 (SPSS, Chicago, Illinois, USA).

## Results

Analysis of the records from the past 13 years in the pathology database of our hospital revealed that only 8 patients with histologically documented prostate cancer with rectal involvement fulfilled the inclusion criteria. After reviewing all medical records, 3 patient was excluded since pathology revealed prostate sarcoma, squamous cell carcinoma, small cell carcinoma respectively. The remaining 5 patients were eligible for the review of the prostate cancer with rectal involvement. After reviewing the 35 articles previously described in “Patient population,” we found 22 suitable articles with clinical description of prostate cancer with rectal involvement. Of these 22 suitable reference articles, 11 case reports [7-10,13-20] (with 28 cases) were in English, 5 in Japanese [21-25] (with 7 cases), and 2 in Portuguese Language [26,27] (with 2 cases), with a total of 37 cases being identified. We also found 3 review articles in Japanese [28-30] (with 47 cases). Thus, 89 cases of prostate cancer invading rectum were identified from the literature. Altogether, data on 94 prostate cancer patients with rectal involvement were gathered for further analysis.

**Figure 1:**
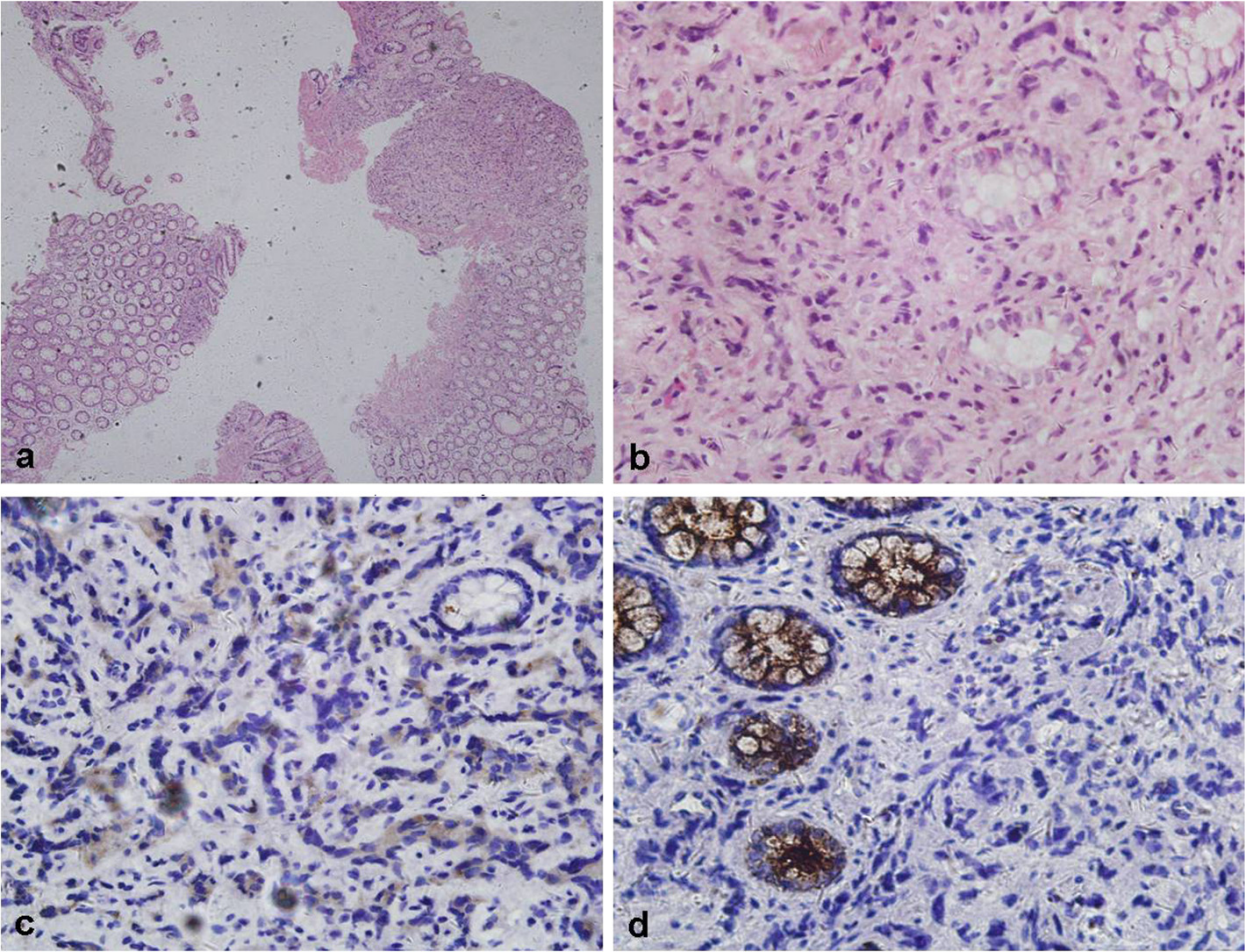
**Rectal biopsy figures show typical prostate cancer with rectal involvement. a** and **b**. Specimens of rectal biopsy show poorly differentiated adenocarcinoma infiltrating rectal mucosa (hematoxylin-eosin, original magnification × 40 and × 400). **c**. Immunohistochemical staining with anti-PSA antibody, showing that tumor cells were positive while normal colonic glands were negative (original magnification × 400); **d**. Immunohistochemical staining with anti-CEA antibody, showing that tumor cells were negative while normal colonic glands were positive (original magnification × 400).

The clinical characteristics of the 5 patients enrolled from our institute are summarized in Table 1. The clinical characteristics of the 94 patients are presented in Table 2. The typical rectal biopsy and image findings of prostate cancer invading rectum was showed in Figures 1 and 2 respectively.

**Table 1:** Clinical summary of the 5 patients from our institute (Tianjin Medical University Cancer Hospital and Institute)

**No.**	**Age (years)**	**Symptoms: bleeding/obstruction/ hydronephrosis/ dysuria**	**PSA (ng/ml)**	**Time to development of RI**	**Type of RI**	**Pathological grade of RI**	**Extrarectal metastases**	**Primary treatment modalities**		**Survival/survival time (months)**
								**Radiotherapy**	**Hormonal therapy**	
1	65	Y/N/N/N	20	S	I	PD	N	Y	Y	DOD/48
2	65	Y/N/N/N	124.5	S	II	PD	Y	N	Y	AWD/18
3	68	Y/N/N/N	3.98	M	III	PD	Y	Y	N	DOD/1
4	80	N/N/N/Y	1050	S	I	MD	Y	N	Y	DOD/30
5	58	N/N/N/N	92.41	S	II	PD	N	Y	Y	AWD/62

Y Yes; N no; RI: rectal involvement; DOD: dead of disease; AWD: alive with disease; S Synchronous rectal involvement at first cancer diagnosis; M Metachronous diagnosis of rectal involvement; PD = poorly differentiated; MD: moderately differentiated; Type of RI was defined according to the criteria described in the literature [11,12].Type I means anterior rectal mass; Type II means annular rectal stricture; Type III means ulcerating anterior rectal mass; Type IV means separate metastasis to rectum.

**Table 2:** Clinical characteristics of 94 prostate cancer patients with rectal involvement (RI)

**Clinical characteristics**	**Synchronous rectal involvement at first cancer diagnosis**	**Metachronous diagnosis of rectal involvement**	**Total**
	**(n = 63)**	**(n = 31)**	**(n = 94)**
Age at rectal involvement	72(57-86)(63)	75(47-94)(27)	72.04(47-94)(90)
PSA at rectal involvement(ng/ml)^#^	113(3.9-7650)	32.75(0.008-1173)	463.10(0.008-7650)
Interval between primary tumor diagnosis and RI(months) & Race	0	40(6-216)	10.5(0-216)
Japan	46	16	62
USA	4	7	11
China	4	1	5
Brazil	2	0	2
France	0	1	1
Qatar	1	0	1
Pathological grade of RI			
well differentiated	2	0	2
moderately differentiated	10	2	12
poorly differentiated	43	20	63
ND	8	9	17
Histology of prostate cancer RI			
Prostate adenocarcinoma	61	29	90
Mixed type	4	2	6
Tumor extent at diagnosis of RI			
RI only	20	5	25(
Extrarectal metastases	28	16	44
ND	15	10	25
Prior therapy			
Hormonal therapy	0	28	28
Initial prostatectomy or radical radiotherapy	0	7	7
TURP	0	4	4
Type of rectal involvement			
I	7	2	9
II	34	10	44
III	8	4	12
IV	3	8	11
ND	12	6	18
Clinical symptoms			
Dysuria	14	4	18
Dyschezia	47	17	64
Hydronephrosis	14	3	17
Rectum bleeding	14	9	23
bowl obstruction	5	3	8
Misdiagnosized before therapy^*^	18	3	21
Treatment modalities after diagnosis of RI			
colostomy	9	10	19
TPE	11	1	12
RR	6	3	9
RT	9	4	13
HT	63	3	66
chemotherapy	0	3	3
TPE + HT^@^	12	0	12
(RR or RT or TURP) + HT	12	0	12

Values are given as median (min-max) or absolute numbers (number of patients).

ND, not documented; RI, rectal involvement; TURP, transurethral resection; TPE, HT, hormone therapy; RT, radiotherapy; RR, rectum resection

# PSA at rectal involvement (ng/ml) were available in 38 patients with synchronous and 16 patients with metachronous diagnosis.

& Interval between primary tumor diagnosis and rectal involvement were available in 25 patients.

*Before therapy, patients were misdiagnosized as rectal cancer, concomitant rectal and prostate cancer, and bladder cancer in 16, 3 and 2 patients respectively.

@ 4 patients were given neoadjuvant HT + TPE + adjuvant HT

“Mixed type” means prostate adenocarcinoma mixed with signet ring cell or ductal adenocarcinoma or papillary carcinoma.

**Figrue 2:**
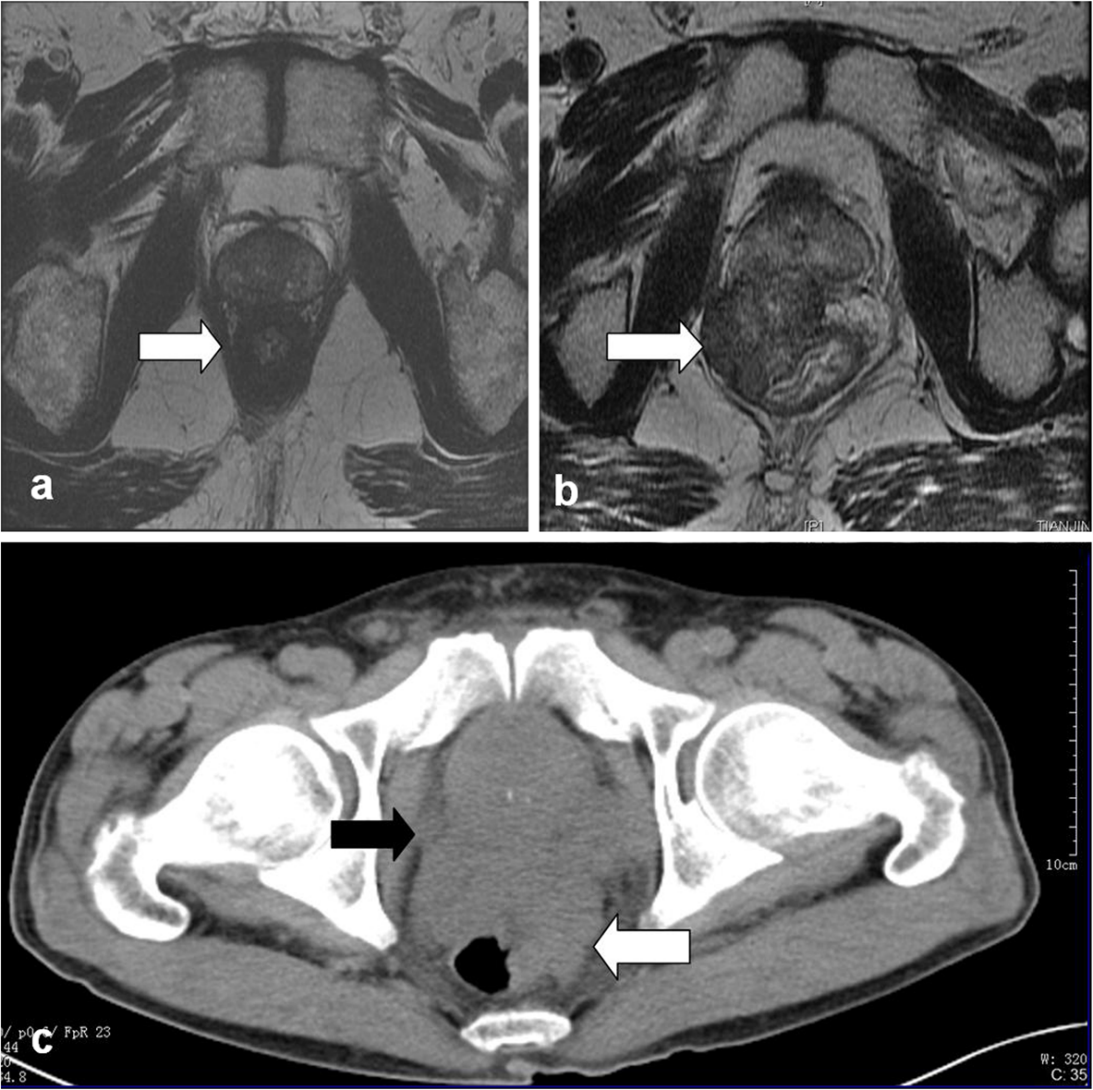
**Typical images show prostate cancer invading rectum. a**. Pelvic MRI showed a circumferential thickening of the rectum, although there was no continuity between prostate and rectum; **b**. Pelvic MRI showed a protruding anterior rectal mass which compressed the rectal lumen and continuity between prostate and rectum; **c**. CT of the abdomen and pelvis showed an irregular thickening of the rectum and continuity between prostate and rectum.

At diagnosis, a high proportion of patients (63.77%) showed extrarectal PCa metastases, and PCa with rectal involvement only were found only in 25 (36.23%) cases. 21 patients (22.3%) were misdiagnosed preoperatively as rectal cancer, concomitant rectal and prostate cancer, or bladder cancer in 16, 3 and 2 patients respectively.

The appropriate treatment was given on an individual basis according to the severity of the clinical symptoms, preoperative diagnosis, organs involved, hormonal status at diagnosis of rectal involvement, patient's age and patients’ preferences. 19 of these patients (20.2%) have accepted diverting colostomy for rectal occlusion. Treatment of primary prostate cancer for the patients in the hormone relapsed group consisted of hormonal therapy, either with luteinizing hormone-releasing hormone (LHRH) agonists or orchiectomy or combined with antiandrogens. 1 patient underwent TURP, and 7 patients received radical prostatectomy or curative radiation therapy. After diagnosis of rectal involvement at hormonally relapsed disease, 3 patients were given chemotherapy. 4 of these patients received palliative radiotherapy due to rectal bleeding or local pain, 1 patient received total pelvic extenteration (TPE) and 3 patients were given abdomino-perineal excision of the rectum because of misdiagnosis as a carcinoma of the rectum.

All of the 63 patients with hormone-untreated prostate cancer were treated with hormonal therapy, either with luteinizing hormone-releasing hormone (LHRH) agonists or orchiectomy or combined with antiandrogens. Among these who underwent hormonal therapy, part of the patients (31.7%) also underwent different types of local therapy, 12 of these patients were given TPE, 9 patients received radiotherapy and rectum resection were given because of misdiagnosis as a carcinoma of the rectum. Among the 12 patients who underwent TPE, 2 died of prostate cancer at 51 and 81 months respectively, 2 died of other disease at 1 and 70 months respectively.5 patients were still alive with disease-free status for 8,10,24,36 and 96 months; 3 patients were still alive with biochemical or clinical progression at 13,13 and 45 months. Interestingly, TPE combined hormonal therapy give us six of thirteen long-term survivors for more than 3 years in this series. Surprisingly, most of these diseases were extrarectal disease, only 1 with rectum only disease. In addition, 6 patients underwent neoadjuvant and adjuvant hormonal therapy, while another 7 patients received adjuvant hormonal therapy.

Of the 94 patients included, data was available on the survival status of 87 patients. The data and the hazard ratios with overall survival of demographics, clinical characteristics, tumor characteristics, treatment for all the 87 cases are shown in Table 3.

**Table 3:** Demographics, clinical and tumor characteristics, treatment modalities associated with overall survival by Cox univariate analysis in 87 patients

	**n (%) or mean ± SD**	**Hazard ratio (95% confidence interval)**	**p value**
Age	72.04 ± 8.427	1.044(0.998-1.093)	0.063
Grouped by age			
<73	37(44.58%)	1	
≥73	46(55.42%)	1.521(0.696-3.326)	0.293
Race			
Non-Asian	24(27.59%)	1	
Asian	63(72.41%)	0.291(0.140-0.606)	0.001
Clinical symptoms			
Hydronephrosis			
No	70(80.4%)	1	
Yes	17(19.54%)	0.904(0.389-2.104)	0.786
Dysuria			
No	69(79.31%)	1	
Yes	18(20.69%)	1.939(0.783-4.805)	0.815
Dyschezia			
No	27(31.03%)	1	
Yes	60(68.97%)	1.193(0.557-2.556)	0.650
bowel obstruction			
No	80(91.9%)	1	
Yes	7(8.1%)	0.821(0.292-2.310)	0.709
Rectum bleeding			
No	67(77.01%)	1	
Yes	20(22.99%)	2.145(1.023-4.498)	0.043
The Time to development of RI			
Synchronous presentation	58(66.67%)	1	
Metachronous presentation	29(33.33%)	14.339(5.719-35.952)	0.000
Tumor characteristics at diagnosis of rectal involvement			
Pathological grade of RI			
well or moderately differentiated	12(17.14%)	1	
poorly differentiated	58(82.86%)	2.048(0.474-8.853)	0.337
Histology of prostate cancer RI			
adenocarcinoma	81(93.10%)	1	
Mixed type	6(6.90%)	0.356(0.048-2.615)	0.310
CEA			
<5 ng/mL	26(83.87%)	1	
≥5 ng/mL	5(16.13%)	2.795(0.510-15.331)	0.237
CA199			
<37 u/mL	23(85.19%)	1	
≥37 u/mL	4(14.81%)	2.150(0.223-20.768)	0.508
PSA	464.74 ± 1189.28	1.000(0.998-1.000)	0.499
PSA			
<92.41 u/mL	28(52.83%)	1	
≥92.41 u/mL	25(47.17%)	0.136(0.153-1.248)	0.122
Type of rectal involvement			
Type I	9(13.04%)	1	
Type II	40(57.97%)	0.494(0.151-1.615)	0.244
Type III	11(15.94%)	1.163(0.311-4.346)	0.822
Type IV	9(13.04%)	1.653(0.433-6.304)	0.462
Hormonal status			
No prior hormonal therapy	61(70.11%)	1	
Prior hormonal therapy	26(29.89%)	14.658(6.042-35.561)	0.000
Tumor extent at diagnosis of RI			
rectum-only	24(35.82%)	1	
Extrarectal metastases	43(64.18%)	2.618(0.984-6.961)	0.054

RI: rectal involvement.

Type of RI was defined according to the criteria described in the literature [11,12].

CEA and CA199 were analyzed as categorical variables using normal value as cut off; Age and PSA were analyzed as categorical variables using median value as cut off.

**Figure 3:**
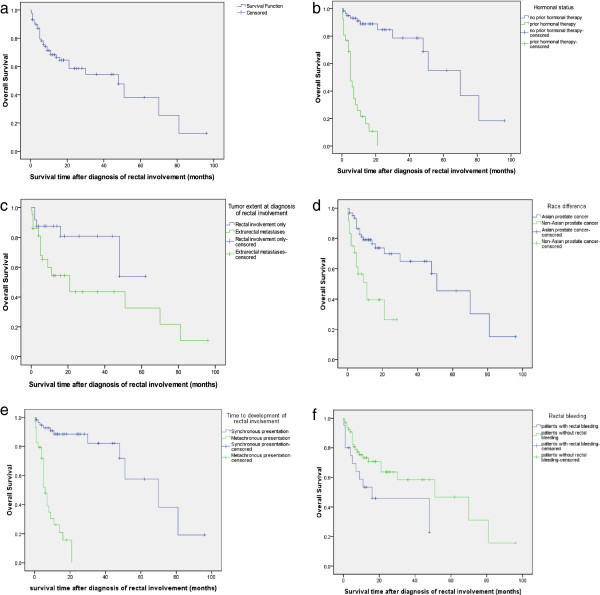
Kaplan-Meier estimates of overall survival (a) and stratified according to (b) hormonal status (Log Rank, p = 0.000); (c) tumor extension (Log Rank, p = 0.041); (d) race differences (Log Rank, p = 0.000); (e) time to development of rectal involvement (Log Rank, p = 0.000); and (f) rectal bleeding (Log Rank, p = 0.036).

11 patients in the group of synchronous rectal involvement at first cancer diagnosis (n = 58) and 23 patients in the group of metachronous diagnosis of rectal involvement (n = 29) died at the latest follow up. Since more than half of the patients are still alive it is not possible to calculate survival accurately, but it is estimated by the Kaplan-Meier method that the median overall survival after diagnosis of rectal involvement will be about 48.0 months (95% CI 22.3-73.7). The estimated overall survival rate (% ± SE) at 1, 3, and 5 years were 68.3 ± 5.3%, 54.4 ± 7.2%, and 38.1 ± 11.1%, respectively (Figure 3a). The univariate associations between overall survival and the clinical characteristics are summarized in Table 3 and Figure 3b-e.The results of the multivariate analysis are shown in Table 4. In Cox univariate analysis, Asian prostate cancer (p = 0.001) was associated with better survival, while rectal bleeding (p = 0.043), metachronous presentation of development of rectal involvement (p = 0.000), prior hormonal therapy (p = 0.000) and extrarectal metastases (p = 0.054) were associated with poor survival. Other variables were not significantly associated with survival. In multivariate analysis, prior hormone therapy (HR = 14.540, 95% CI 5.978-35.361; p = 0.000) and rectal bleeding (HR = 2.195, 95% CI 1.035-4.655; p = 0.041) retained independent poor prognostic values.

**Table 4:** Multivariable analysis with Cox regression model to determine independent prognostic factors for overall survival in 87 prostate cancer patients with rectal involvement

	**Hazard ratio (95% confidence interval)**	**p value**
Synchronous versus metachronous presentation		0.109
Prior hormone therapy (Yes versus No)	14.540(5.978-35.361)	0.000
Rectal bleeding (Yes versus No)	2.195(1.035-4.655)	0.041
Country (Asian versus Non-Asian)		0.187

## Discussion

Prostate cancer is the second most common cancer in men with metastasis commonly occurring in the bones [6]. Advanced prostate cancer may spread out of the prostate and involve adjacent pelvic organs, such as the seminal vesicle, urinary bladder, and rectum. Of these, the rectum is the least commonly involved by prostate cancer. Rectal involvement, by direct invasion or metastasis, occurs in 1.5% to 11% of those with prostate cancer [11,12,31,32]. The possible reason for rectal involvement may be related to tumor’s biological behavior such as gene aberration or tumor dedifferentiation, positive surgical margin or collision tumour in the very rare situation [33-35]. The infrequent spread to the rectum despite its close proximity to the prostate gland has been attributed to Denonvilliers’ fascia, a critical barrier to local extension of tumor [36,37]. The studies dealing specifically with rectal involvement from prostate cancer are rare and have not been well reported. To our knowledge, this is the first report to investigate predictive factors of survival in prostate cancer with rectal involvement. The results of our study are thus important for physicians to guide treatment selection and to counsel patients about their long-term outlook in prostate cancer with rectal involvement.

The diagnosis of prostate cancer with rectal involvement is easily made by rectal examination, radiologic characteristics, and especially histological examination of the rectal biopsy in conjunction with the level of prostate specific antigen. However, occasionally, prostate cancer with rectal involvement may be confused with a primary rectal carcinoma because of similar radiologic appearances and clinical presentations such as large-bowel obstruction and rectal bleeding, etc. Correct diagnosis is essential since the treatment and prognosis of these two entities vary significantly. In the present study, 21 patients (22.34%) were misdiagnosed preoperatively as rectal cancer, concomitant rectal and prostate cancer, or bladder cancer in 16, 3 and 2 patients respectively. The definitive diagnosis was finally established by postoperative histopathological work-up. In these cases, the preoperative misdiagnosis poses the therapeutic decision-making a greatest problem. Although the levels of PSA, radiographic appearance, and clinical presentation may be helpful in distinguishing the two entities, histological examination of the lesion may be the only method of making the correct diagnosis. It may be difficult to differentiate rectal adenocarcinoma from prostatic adenocarcinoma in small rectal biopsy specimens, although it is generally not a problem in operation specimens [38,39]. The morphological features that favor rectal adenocarcinoma include ‘dirty’ necrosis, tall columnar cells with basally located nuclei, mucin production, villous architecture and stromal inflammation. When a definitive diagnosis cannot be established on the basis of morphological characteristics, immunohistochemical studies can be helpful. Rectal adenocarcinoma, unlike prostatic adenocarcinoma, shows positive staining for b-catenin, caudal-related homeobox 2 (CDX2) and carcinoembryonic antigen(CEA) but negative staining for PSA, PSAP, P501S [40,41].

Traditionally, prostate carcinoma presenting with rectal involvement represents an advanced stage of a highly invasive form of the disease. The prognosis is poor. Bowrey et al. retrospectively described the outcome of six prostate cancer patients with rectal infiltration and reviewed the literature for 1966–2002. The median survival for the 86 patients with outcome data reported was 15 months (95% CI 14–16 months). Survival beyond 30 months was rare [10]. While, in the present study, Kaplan-Meier analysis estimated that the median overall survival was 48 months. There were 13 patients survived for more than 3 years, the longest survival time was 96 months. This apparent discrepancy from previously published data may be attributed to distinction rarely made according to the hormonal status at presentation, different follow up time, race differences of prostate cancer, also advancement of treatment and diagnosis.

As expected, hormonal status at presentation had a major impact on overall survival in PCa patients with rectal involvement on both univariate and multivariate analysis. In the present study, patients previously untreated by hormones had a relatively good prognosis with an estimated median survival of 70 months (95% CI, 33.154-106.847), the estimated 1, 3, 5 year survival rate were 88.9%, 78.6% and 55.0%, respectively, and half of them were still alive at the latest follow-up, which is at least equivalent to patients presenting with minimal or extensive metastatic prostate cancer treated with androgen deprivation in large series [42]. Patients presenting with hormone relapsed disease had a much poorer outcome with a median survival of 5 months (95% CI, 3.456-6.544). Therefore, rectal involvement does not necessarily predict a worse outcome when presented as hormone untreated disease; when presented as a hormonally relapsed disease, prognosis was worse. The possible reason is that those with previous hormonal therapy have no or few effective options left after RI occurs. In the context of an increasing pipeline of novel approved agents (abiraterone acetate, sipuleucel-T) and emerging agents (radium-223, MDV-3100), optimal survival will probably be realized by the sequential utilization of several different classes of agents.

Rectal involvement comprises a multitude of differing clinical presentations. Unless one patient present asymptomatic during a routine screen for occult metastases, all other patients present with specific symptoms. In our series, patients presented with rectal bleeding had significantly poor survival on univariate analysis (p = 0.043). Furthermore, the presence of rectal bleeding is an independent prognostic factor predicting for worse prognosis (HR 2.195, p = 0.041). It is unclear whether the tumors of patients presenting with rectal bleeding have a different biology. Bleeding may indicate tumors of larger luminal surface, erosions, or highly vascular tumors.

An interesting finding from this study is that Asian patients appeared to predict better overall survival than non-Asian patients on univariate analysis (p = 0.001). The results support a previous report that many Asian prostate cancer patients had better survival than whites [43]. The survival advantage for Asian patients may be due to factors not measured in our study, such as endogenous hormone levels, dietary factors, or body composition. Further research is needed to identify which, if any, of these factors may explain these obscure findings for Asian men. All these factors are particularly difficult to study because it is unclear, for example, which of the dozens of hormones involved in testosterone metabolism should be measured, and at what age to measure. Understanding the potential interactions of each of these factors will be critically important for the development of effective prevention strategies.

Rectal involvement are often a sign of more widespread systemic disease, considering the sixty-four percent of patients had extrarectal metastases at the diagnosis of rectal involvement. Patients with extrarectal metastasis disease were associated with poor overall survival in comparison with patients who had singly rectal involved disease (Log Rank, p = 0.041; Cox, p = 0.054). Multivariate analysis was not performed because of the incomplete follow up information for this covariate, so whether it is an independent prognostic factor deserves further evaluation. However, Stage IV subcategory has been identified as independent predictor of overall survival in stage IV prostate cancer [44]. Prostate cancer patients with stage cT4N0M0 had significantly better overall survival on multivariate analysis compared with stageTxNxM1 patients (p < 0.001).

The role of curative surgery in treating prostate cancer patients with rectal involvement has been controversial. Historically, TPE has been generally unsuccessful when used as a curative approach to treat locally advanced prostate cancer in several small-sample case series and the role of TPE was limited to palliate debilitating perineal pain and other local symptoms, such as haematuria, urinary obstruction, and urinary and rectal incontinence [45]. Spaulding and Whitmore reported no long-term survivors among patients with locally advanced prostatic adenocarcinoma involving the rectums who were treated with total pelvic exenteration [46]. Similarly, Zincke reported on 7 patients who underwent total pelvic exenteration after failure of radiation therapy [47]. Despite the extensive local procedure, the residual cancer rate was high and local control was not improved with a median time to progression of 15 months. Many articles have cited these findings and advocated a less invasive treatment such as external beam radiotherapy [45,48]. In this retrospective series, for the hormonally untreated patients, there were three treatment strategies based on hormonal therapy.19 patients (31.14%) have been treated with hormonal therapy only, other 25 patients (40.98%) have been treated with combining hormonal therapy with radiotherapy or rectum cancer operation because of the misdiagnosis as rectum. Surprisingly, the remaining 12 patients were given TPE (19.67%). The comparison of survival differences between the three treatment groups could not be done because of the high censoring rate with short follow up in these patients.However, interestingly, in this series, TPE combined with hormonal therapy in 12 patients give us six of thirteen long-term survivors for more than 3 years with the longest survival time of 96 months. Furthermore, 5 patients were still alive with disease-free status for 8, 10, 24, 36, 96 months. The above observations indicated that TPE combined with hormonal therapy appears to confer better survival for hormonally untreated prostate cancer patients with rectal involvement.It is also important to note that 9 of the 12 had extrarectal metastases at the time of surgical resection. This indicated that extrarectal spread of disease is not a contraindication to TPE and that such patients may also attain long-term survival. In addition, we believe that TPE may also provide potential survival benefit in hormonally relapsed prostate cancer with rectal involvement only disease, because a study [45] showed the median overall survival time of 24 months comparing with 5 months of this series. What is the underlying reason for TPE to improve survival in prostate cancer patients with rectal involvement? The possible explanation is local control, removing the potential source of future metastases, and improving the response to systemic therapy [44]. Although it is inappropriate to make inferences regarding the role of TPE in the management of prostate cancer with rectal involvement based on the findings in this retrospective review, we and others [49] believe that TPE should be further evaluated as treatment strategies for selected patients, especially in nonextrarectal metastatic hormonally untreated patients, because they may provide better local control and a potential long-term survival and cure.

This study had several limitations. Due to the extreme rarity of prostate cancer with rectum involvement, we could obtain the data for only 5 patients who were admitted over the last 13 years to our hospital. Hence, we collected all available cases from the literature and found an additional 89 cases. Because of the retrospective nature of data collection, the limited information provided in the literature, and short follow-up period with high censoring rate, the statistical results should be interpreted cautiously. Therefore, the absence of association of some factors with survival could be attributed to the small case numbers which may result in insufficient statistical power. Nevertheless, the findings of this study are still valuable for better understanding the disease processes of prostate cancer with rectal involvement and the factors affecting the survival of patients with this rare disease.

In conclusion, our findings suggest that rectal involvement does not necessarily predict a worse outcome when presenting as a previously hormone-untreated disease and that the prognosis was worse when presenting as a hormonally relapsed disease. Although limited by the short follow-up period with high censoring rate, our study indicates that prior hormone therapy and rectal bleeding were independently associated with a significantly poor overall survival in prostate cancer patients with rectal involvement. TPE combined with hormonal therapy appears to confer better overall survival in hormonally untreated patients. It is also necessary to determine who can benefit from TPE for prostate cancer with rectal involvement patients.

## Competing interests

The authors declare that they have no competing interests.

## Authors’ contributions

HTW and BGL: acquired the data and draft the manuscript. HTW and YHY performed the statistical analysis. All authors read and approved the final manuscript.
